# Circulating Liver-Specific miR-122 as a Novel Potential Biomarker for Diagnosis of Cholestatic Liver Injury

**DOI:** 10.1371/journal.pone.0073133

**Published:** 2013-09-27

**Authors:** Huang Shifeng, Wang Danni, Chen Pu, Yang Ping, Cao Ju, Zhang Liping

**Affiliations:** Department of Laboratory Medicine, the First Affiliated Hospital of Chongqing Medical University, Chongqing, China; Sapporo Medical University, Japan

## Abstract

**Background:**

Circulating microRNA-122 (miR-122) has been increasingly reported to be a potential biomarker for drug-, viral-, alcohol- and chemical-induced liver injury. The present study was initiated to determine the potential of circulating miR-122 as a biomarker for cholestatic liver injury.

**Methods:**

Both bile-duct ligation (BDL) mice and patients with biliary calculi were employed as cholestatic liver injury models, and serum miR-122 level was determined by stem-loop real-time reverse-transcription PCR (SLqRT-PCR). All quantitative PCR values were normalized to those for U6 RNA and calculated with the 2^−△Ct^ method.

**Results:**

Serum miR-122 increased significantly after BDL-induced cholestatic injury and showed a similar time course to ALT concentrations. Compared with the sham controls, BDL mice had increased serum levels of miR-122 by 24.36±12.86, 423.63±322.89, 4.43±2.02 and 12.23±8.92 folds after 1, 3, 7 and 14 days, respectively. Moreover, serum miR-122 level was substantially higher in patients with biliary calculi than that in the healthy control group. In addition, patients with severe liver injury showed significantly higher levels of serum miR-122 when compared with healthy controls or patients with mild or moderate liver injury. Furthermore, serum miR-122 was found to show significant diagnostic value for biliary calculi by yielding an AUC (the areas under the receiver operating characteristic curve) of 0.931 with 77.4% sensitivity and 96.4% specificity in discriminating biliary calculi from healthy controls.

**Conclusion:**

Collectively, these data suggest that serum miR-122 has strong potential as a novel, specific and noninvasive biomarker for diagnosis of cholestasis-induced liver injury.

## Introduction

Cholestasis-induced liver injury, a frequent clinical event, has an enormous economic impact on health care expenditures. While there is a strong correlation between stage and prognosis in cholestasis, current screening methods for cholestaic liver injury have significant limitations, and the exploration of new biomarkers with high sensitivity and specificity in early diagnosis of cholestasis never stops.

miRNAs are single-stranded RNAs of endogenous origin that post-transcriptionally regulate gene expression, typically by mediating mRNA degradation and/or translational blockade after binding to complementary sequences in the 3’ nontranslated region (3’UTR) of the target mRNA [1], and thereby play important roles in a wide range of physiological and pathologic processes [2], [3]. Of note, miRNAs are emerging as important players in liver health and disease, and an involvement of miRNAs was demonstrated in hepatocyte apoptosis [4], obstructive jaundice [5], liver fibrosis [6] and hepatocarcinogenesis [7], [8].

Recently, circulating microRNAs (miRNAs) have opened up a new field for molecular diagnosis of cancer [9], [10] and acute myocardial infarction [11], [12]. More importantly, studies have shown a modulation of serum miRNAs in rodent models of acetaminophen overdose- [13], D-galactosamine- and alcohol-induced liver injury and in patients with viral-induced liver injury [14]. In light of these findings, we hypothesized that circulating miRNAs may reflect liver damage and thus may be regarded as biomarkers of the disease at least under some pathological settings.

One premise for using circulating miRNAs to diagnose disease is the notion that the abundance of the miRNAs in body fluids reflects their abundance in the abnormal cells causing the disease. As a result, the search for such diagnostics in body fluids has focused on miRNAs that are specific or abundant in the cells of origin. Indeed, many miRNAs exhibit a tissue-specific distribution [15] and they appear to play a key role in cell function both under physiological and pathological conditions. MiR-122 was identified as a well-conserved and the most abundant liver-specific microRNA, constituting 70% of total hepatic microRNA [16]. MiR-122 was implicated in regulation of fatty-acid and cholesterol metabolism [17], amplification of hepatitis C virus (HCV) genome [18], response to interferon treatment of patients infected with HCV [19], and carcinogenesis of hepatocellular carcinoma [20]. Thus, it is reasonable to conceive that miR-122 can be a potentially novel biomarker, modulator and therapeutic target for liver diseases. Indeed, recent studies have demonstrated a remarkable antiviral effect in chimpanzees following therapeutic silencing of miR-122 by administration of a locked nucleic acid (LNA) antisense oligonucleotide [21], and miR-122 mimetic alone or in combination with anticancer drugs were demonstrated to be a promising therapeutic regimen against liver cancer [20]. More importantly, circulating miR-122 was recently confirmed as a sensitive and early marker for drug- [13], viral-, alcohol- and chemical-induced liver injury [14]. However, the diagnostic value of circulating miR-122 in other types of liver injury, such as cholestatic liver injury, in animal models and patients remains undefined.

We hypothesized that the level of liver-specific circulating miR-122 may also be used to detect and monitor the pathological development associated with cholestasis-induced liver injuries. Using a BDL mouse model and clinical patient samples, we investigated the utility of miR-122 as a novel potential serum biomarker for early detection of cholestatic liver injury. We report here for the first time that the serum level of miR-122 is associated with liver injuries induced by cholestasis, and that miR-122 may serve as a potential novel and reliable blood biomarker for noninvasive cholestasis diagnosis.

## Materials and Methods

### Ethics Statement

This study was carried out in strict accordance with the recommendations in the Guide for the Care and Use of Laboratory Animals of Chongqing Medical University. The protocol was approved by the Committee on the Ethics of Animal Experiments of the first affiliated hospital of Chongqing Medical University (Permit Number: 2010-[17]). All surgery was performed under sodium pentobarbital anesthesia, and all efforts were made to minimize suffering.

The protocol of the human research part was carried out according to the 2008 Declaration of Helsinki and approved by the Medical Ethics Committee on human research in the First Affiliated Hospital of Chongqing Medical University (Permit Number: 201103). Written informed consent was obtained from all the participants before enrollment.

### Animals and the construction of BDL mouse model

Twenty-eight 4- to 6-week-old Balc/C female mice (Central Laboratory of animal facility, Chongqing Medical University, Chongqing, China) were maintained at the Animal Facility of Chongqing Medical University. Animals were kept in filter-top cages on sterile bedding and provided with sterile food and acidic water ad libitum. Cholestatic liver injury was induced by bile-duct ligation, as described previously [22]. Sham-operated mice, used as controls, underwent a laparotomy with exposure, but no common bile duct ligation was performed. Lliver tissues and blood samples were drawn before the operation and at 1, 3, 7 and 14 d after the ligation.

### Serum collection and storage

In our experiments, serum of human patients from an independent group of 31 patients with biliary calculi and 28 healthy control individuals were collected for biomarker-validation. A cohort of 31 patients with biliary calculi who had undergone imageological measurement was enrolled which were characterized with localisation of calculi gallbladder, bile duct, biliary pancreatitis or cholangitis. Patient blood samples for miRNA detection were collected from 31 consecutive biliary calculi with right upper quadrant abdominal pain admitted to Department of emergency and 28 healthy volunteers (normal liver function finding and no history of hepatobiliary disease) underwent routine physical examination in the First Affiliated Hospital of Chongqing Medical University between Jan 2011 and Feb 2011.

Blood samples from both patients and mice were centrifuged within 1 h of collection, and the serum was transferred to RNase/DNase-free tubes and stored at −80°C.

### Serum chemistry

Standard automatic biochemistry analyzer (OLYMPUS AU5400) was employed to detect the levels of ALT, AST, ALP, GGT, DBIL and TBIL in serum obtained from BDL and sham-operated mice.

### Histological and pathological assay

Liver tissues were sliced into 5×5-mm sections, fixed in 4% paraformaldehyde for 48 hours, and then embedded in paraffin. Tissue sections were prepared with a microtome at 5 µm and placed on glass slides. Liver mitosis and inflammatory infiltration were observed microscopically after hematoxylin and eosin (HE) staining.

### Immunohistochemistry

For immunohistochemical analysis, sections were first stained for CK-19 using goat polyclonal antibodies (Santa Cruz Biotechnology, Santa Cruz, CA, USA), followed by a second reaction with biotin-labeled anti-goat IgG. Peroxidase activities were visualized by applying diaminobenzidine to the sections, which were then counter stained with haematoxylin.

### Serum miRNA isolation

Total RNA enriched with miRNAs was isolated from serum using the mirVana PARIS miRNA isolation kit (Amibion) according to the manufacturer’s instructions.

### Stem-loop real-time RT-PCR

To determine the expression level of miR-122, stem-loop real-time RT-PCR (SLqRT-PCR) was performed. miRNAs were quantified by using TaqMan miRNA quantitative reverse transcriptase–polymerase chain reaction (qRT–PCR) assay according to the protocol of the manufacturer (Applied BioSystems, Inc.). Briefly, total RNA (10 ng) was used for first-strand cDNA synthesis using miRNA-122-specific, stem-loop primer, or U6 stem-loop primer, a control endogenous miRNA (Applied Biosystems, Foster City, CA), followed by real-time PCR amplification with gene-specific forward primer and a reverse primer along with a probe, in an ABI Prizm 7500 PCR machine. The relative miRNA expression was calculated from three different experiments. All reactions were run in triplicate, and results were normalized to those for U6 RNA. Relative miR-122 production was determined with the △Ct method and reported as 2^−△Ct^ , where Ct represents the threshold cycle. Differences in miR-122 concentration in the disease group compared with the control group were expressed as fold changes.

### Statistics

Unless otherwise indicated, the mean and SD were calculated. Differences between 3 or more groups were analyzed with the Kruskal–Wallis test. Between-group comparisons were made with the Mann–Whitney U-test, Pearson χ2 test, Student t-test, and Spearman correlation analysis as appropriate. Values obtained at baseline (0 d) were used as the reference and compared with those obtained at 1, 3, 7, and 14 d after BDL or sham operation. To assess whether the time courses of miR-122 and ALT, AST, ALP, DBIL and TBIL differed, we normalized these values and compared them by multivariate ANOVA. ROC curves were established for discriminating patients with cholestatic liver injury from the normal control ones. Two-tailed P values<0.05 were considered statistically significant. All statistical analyses were performed with SAS software (version 9.1.3; SAS Institute) and SPSS software (version 17.0).

## Results

### Characterization of inflammation, biliary epithelial cellular proliferation and hepatobiliary dysfunction in BDL mice

Microscopically, HE staining of liver sections showed that inflammatory cells such as neutrophils were infiltrated into hepatic tissues of the BDL mice (Figure S1 B to E), while no neutrophil infiltration was found in the sham-operated group (Figure S1 A). The extravasated neutrophils will probably create a positive feedback loop in which increased liver injury leads to increased neutrophil infiltration, and the infiltrating neutrophils will cause increased liver injury.

To confirm the epithelial immunophenotype, the expression of CK-19, a specific biliary proliferation marker, was investigated in mice liver sections. While there was an induction of CK-19 staining in ductal cells after BDL for 2 weeks (Figure S2 B), no apparent stainings were observed in sham-operated mice livers as expected (Figure S2 A).

To investigate hepatobiliary dysfunction after BDL, serum alanine aminotransferase (ALT), aspartate aminotransferase (AST), alkaline phosphatase (ALP), γ-glutamyl transpeptidase (GGT), direct bilirubin (DBIL) and total bilirubin (TBIL) were determined (Table S1). There was a dynamic change of serum ALT in BDL mice, reaching its peak at day 3 after BDL; meanwhile, serum AST, ALP, TBIL and DBIL were found to be progressive increased during BDL, indicating severe obstructive cholestasis happening in BDL mice model. Taken together, these data indicated impaired liver function in BDL mice when compared with the sham-operated ones.

### Serum miR-122 could be used to detect cholestatic liver injury in BDL mice and showed a similar time course to ALT

**Figure 1:**
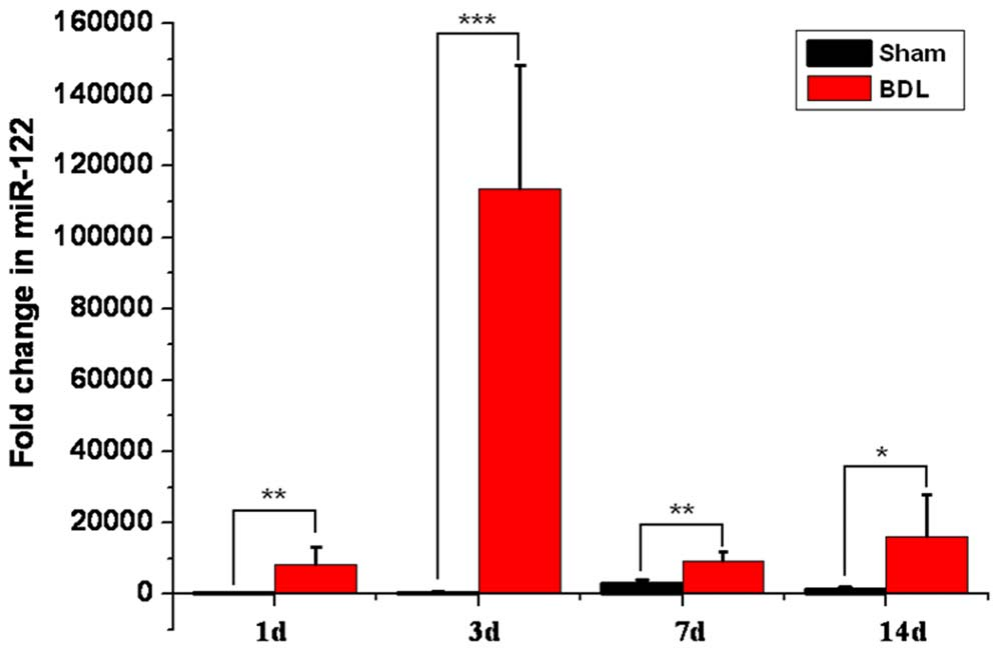
Increased serum miR-122 concentrations in BDL mice. The values of miR-122 fold change are the average of 5 independent samples from each time point, and the standard derivations are shown as error bars. Data are presented as the mean and SD. *P<0.05, and **P <0.005, Student t-test with Bonferroni correction.

Mice underwent BDL were sacrificed and the serum concentrations of ALT, AST, ALP, TBIL and DBIL were determined and miR-122 were measured. As is shown in Figure 1, BDL-induced miR-122 change was readily apparent, and serum miR-122 level was significantly increased as early as 1 d after administration (P<0.01). Moreover, when compared with the increases in aminotransferase activities in the blood (Table S1), the change in miR-122 concentration appeared more significant (Figure 1). Analysis of miR-122 revealed that compared with the sham-operated control ones, BDL mice had increased serum levels of miR-122 by 25.50±4.65 folds, 287.02±12.68 folds, 3.21±0.21 folds and 16.12±2.76 folds after 1, 3, 7 and 14 days, respectively. An improvement of about one order of magnitude was achieved in 3 day after BDL over all the above mentioned liver-injury indexes.

**Figure 2:**
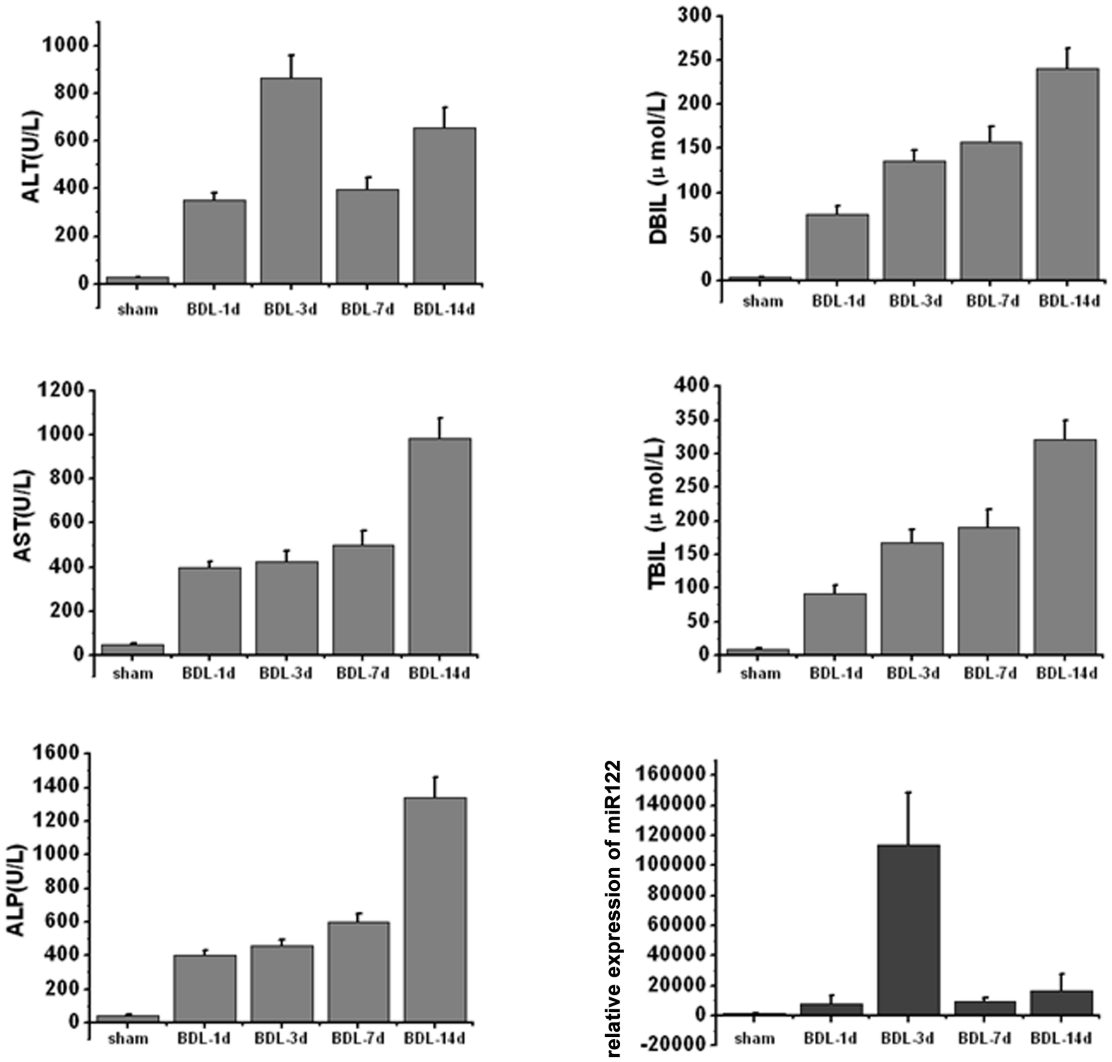
Time courses of serum concentrations of miR-122, ALT, AST, ALP, TBIL and DBIL after BDL. The values of miR-122 fold change and ALT, AST, ALP, TBIL and DBIL levels are the average of 5 independent samples from each time point, and data are presented as the mean and SD.

**Figure 3:**
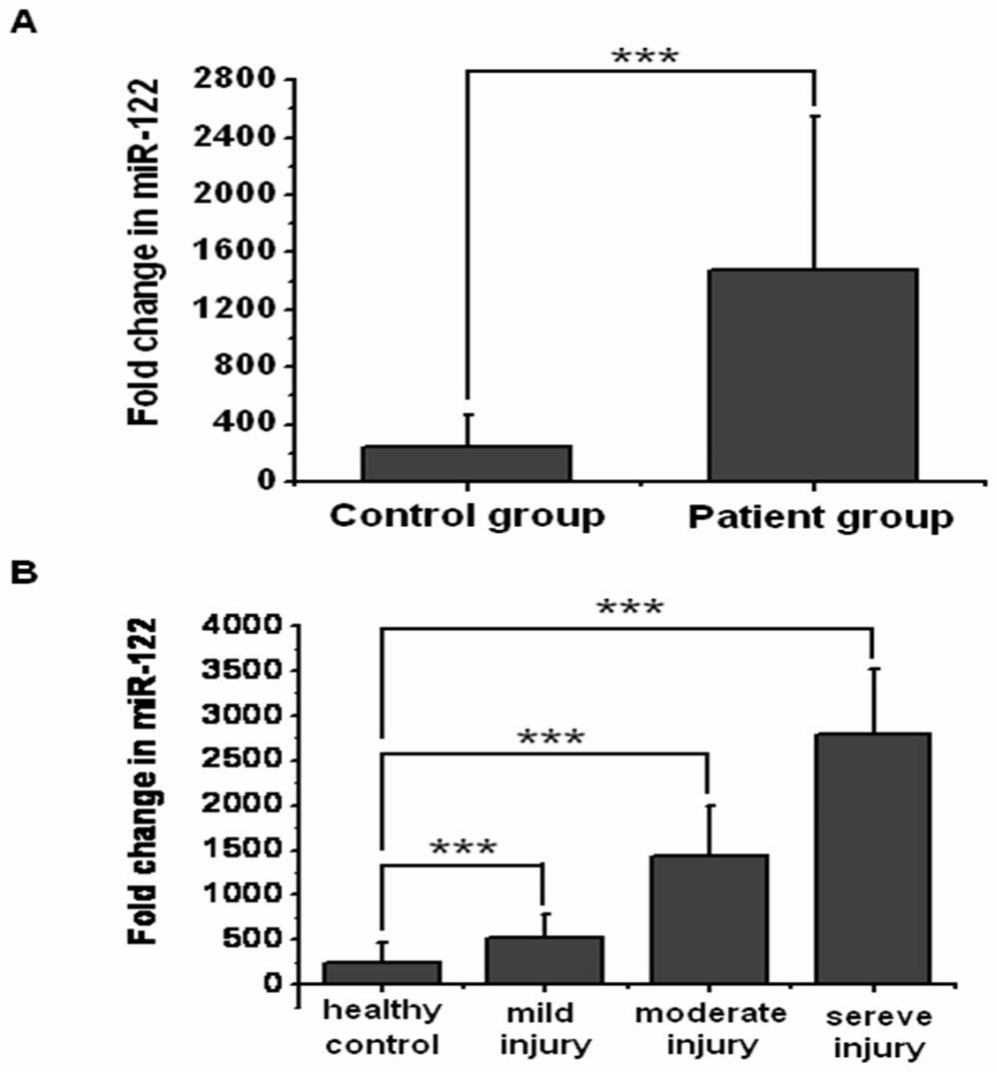
Increased serum miR-122 concentrations in patients with cholestatic liver injury. (A) Comparison of miR-122 concentrations between healthy controls and patients; (B) Serum miR-122 concentrations in correlation with the liver injury degree (mild injury, 80 U/L<ALT≤100 U/L, n = 12; moderate injury, 100 U/L<ALT≤300 U/L, n = 10; severe injury, ALT>300 U/L, n = 9). ***P<0.001.

To better understand the time-course of serum ALT, AST, ALP, TBIL, DBIL and miR-122 levels during BDL, 20 mice were randomly grouped into 4 groups with 5 mice in each group. The animals were then killed at the indicated time points, and serum samples were collected. While the AST, ALP, TBIL and DBIL levels show a time-dependent increase during the tested time duration (Table 1 and Figure 2), the ALT levels didn’t show a time-dependent change, especially in samples from 3 d after treatment. On the other hand, 50 ng of total RNA isolated from each serum sample was used to generate cDNA for miR-122 SLqRT-PCR assay, and data from miR-122 were presented together with ALT, AST, ALP, TBIL and DBIL levels (Figure 2) to explore the similarity in changes between these 2 different types of biomolecules during the progression of BDL-induced cholestatic liver injury. By showing a significant increase 1 d after BDL(P<0.01; Figure 1), reaching its peak at day 3 (P<0.001; Figure 1) and remaining significantly increased until 14 d after BDL (P<0.01; Figure 3), serum miR-122 was found to be dynamically changed during BDL-induced cholestatic liver injury in a time-course that was similar to ALT (Table S1 and Figure 2), a classical biomarker of liver injury. Thus the serum miR-122 may be employed as a useful indicator of cholestatic liver injury. The increase in serum concentration for miR122 was earlier and orders of magnitude higher than other variant. Therefore, we speculated that miR-122 was a more diagnostically sensitive marker for detecting cholestatic liver injury at either end point.

**Table 1:** Clinical characteristics of the healthy control and patient group.

Parameters	control group (n = 28)	Patient group (n = 31)	*P*
Age	57.14±12.69	58.26±14.84	>0.05
Male	17(60.7%)	16(51.6%)	>0.05
Female	11	15	
ALT (U/L)	24.32±6.66	172.45±143.03	<0.05
AST	22.36±5.50	188.62±172.35	<0.05
ALP	93.68±14.95	380.90±369.12	<0.05
GGT (U/L)	25.25±5.70	522.94±441.47	<0.05
TBIL ( µmol/L)	9.53±4.04	83.44±103.65	<0.05
DBIL ( µmol/L)	4.04±1.27	65.24±86.52	<0.05
MiR-122	241.88±218.87	1476.44±1066.14	<0.001

### Serum miR-122 was increased in patients with biliary calculi and showed a significant diagnostic value for cholestatic liver injury

Extending our experiments to human patients, serum from an independent group of 31 patients with biliary calculi and 28 healthy control individuals were collected for biomarker-validation. The general clinical characteristics of the healthy control and the patient group were shown as in Table 1 and 2. While no significant differences were found in age and sex between the patient and the control group, significant differences were found in ALT, AST, ALP, GGT, TBIL and DBIL between the two groups (Table 1).

**Table 2:** Distribution of patients based on clinical feature.

Clinical feature	Cases (n = 31)
calculus of gallbladder	8
calculus of bile duct	7
cholecystolithiasis with	4
cholecystolithiasis with	7
obstructive jaundice	5

The level of serum miR-122 was found to be substantially higher in biliary calculi group than that in the control group (P<0.001, Figure 3 A). To determine whether the serum miR-122 concentration was correlated with the liver injury degree, we evaluated 31 patients and 28 healthy controls and compared the results with the corresponding ALT results. We divided the patients into 3 groups (mild liver injury group, 40 U/L<ALT≤100 U/L, n = 12; moderate liver injury group, 100 U/L<ALT≤300 U/L, n = 10; severe liver injury group, ALT>300 U/L, n = 9 [23]–[25]). miR-122 concentrations were shown to be changed significantly across the 31 patients in the 3 subgroups (P<0.001, Figure 3 B). Moreover, patients with severe liver injury were found to show significantly higher levels of serum miR-122 when compared with healthy controls or patients with mild or moderate liver injury.

**Figure 4:**
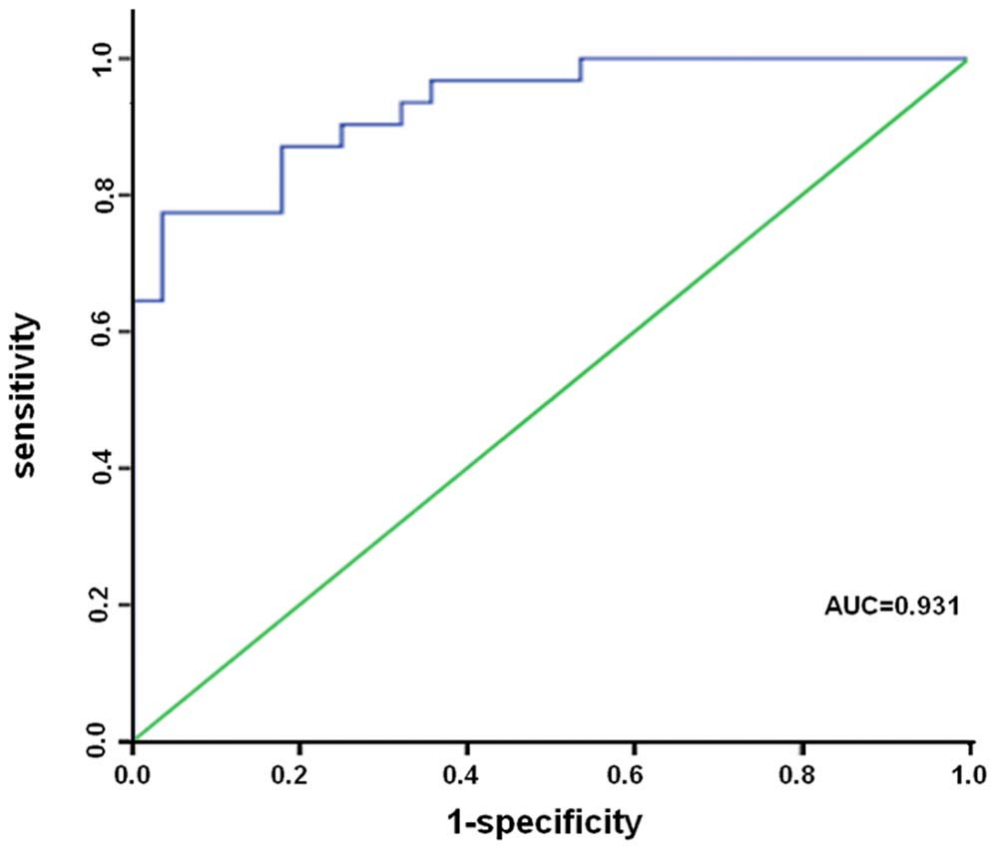
ROC curve analysis of serum miR-122 concentration for discriminating cholestatic liver injury in human patients.

**Figure 5:**
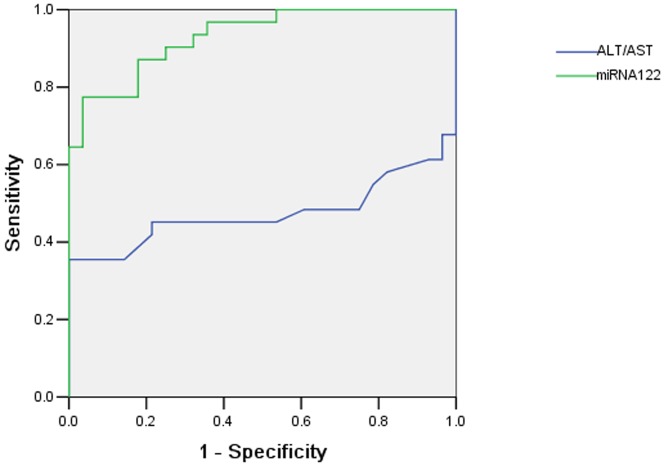
Comparative ROC analysis for miR-122 and ALT /AST for discriminating cholestatic liver injury in human patients.

To investigate the characteristics of miR-122 as a potential biomarker of cholestatic liver injury, we performed ROC curve analysis on data from all 59 samples (31 patients and 28 controls). Serum miR-122 was found to show significant diagnostic value for biliary calculi by yielding an AUC of 0.931 (95% CI, 0.871–0.991; Figure 4) with 77.4% sensitivity and 96.4% specificity in discriminating biliary calculi from healthy controls at a cut-off value of 659.28. As shown as in Fig. 5, the AUC of ALT/AST was 0.475 (95% CI, 0.312–0.637). The results indicated that miR-122 as a potential biomarker of cholestatic liver injury was better than ALT/AST.

## Discussion

Due to the lack of reliable and predictive markers to detect the early signs of cholestasis-induced liver injury, treatment for cholestasis is usually delayed. In the present study, we have demonstrated that serum miR-122 increased significantly during BDL-induced cholestatic liver injury and exhibited a similar time course to the concentration of ALT, a classical biomarker of liver injury. Moreover, compared with the increases in aminotransferase activities in the blood, the change in miR-122 concentration appeared more significant, and an improvement of about one order of magnitude was achieved over the current liver-injury indexes. Furthermore, the clinical relevance was noted by the observation that serum miR-122 levels were enhanced significantly in patients inflicted by biliary calculi relative to corresponding healthy controls. More importantly, patients with severe liver injury showed significantly higher levels of serum miR-122 when compared with healthy controls or patients with mild or moderate liver injury. Finally, serum miR-122 was found to show significant diagnostic value for biliary calculi by yielding an AUC of 0.931 with 77.4% sensitivity and 96.4% specificity in discriminating biliary calculi from healthy controls. These findings suggest the potential of using liver-specific circulating miRNAs as sensitive and informative biomarkers for cholestatic liver injury.

Ever since the cloning of miR-122 from the liver by Lagos-Quintana et al. [26], numerous attention has been focused on trying to understand the functions of this developmentally regulated liver-specific microRNA [27]. Among the predicted targets of miR-122 are factors involved in differentiation, cell cycle progression, inflammation, transcription, protein biosynthesis, cholesterol, and carbohydrate metabolism [28]. In the present study, we serendipitously identified serum miR-122 as a potential novel biomarker for cholestatic liver injury while profiling microRNAs in a rodent model of BDL-induced cholestasis. Extending this study to human patients showed that the up-regulation of serum miR-122 correlates with a severe liver injury in patients with biliary calculi.

Compared with an increase in aminotransferase activities in the blood, the change in miR-122 concentration appeared more significant after BDL. Analysis of miR-122 by use of the mirVana qRT-PCR miRNA detection assay revealed that compared with the sham-operated control ones, BDL mice had increased serum levels of miR-122 by 287.02±12.68 folds at 3 days after BDL, achieving an improvement of about one order of magnitude over all the current liver-injury indexes. In addition, our data demonstrated that the serum concentration of miR-122 showed a good correlation with that of ALT, a classical marker of liver injury, thus clearly supporting the hypothesis that miRNAs may leak out of injured cells into the circulating blood and thereby serve as markers for identifying the type of injured cell.

We further determined miR-122 levels in serum from healthy control and cholestatic patients. Our result that miR-122 was detected at a quite low level in serum from healthy people, but could be easily detected in serum from cholestatic patients revealed for the first time that monitoring the serum levels of miR-122 could also be applied in clinical diagnosis of cholestasis. However, compared with the result from the BDL animal model, we noticed that the serum levels of miR-122 in cholestatic patients were much lower than those in BDL mice. Through ROC analysis, the present work has led us to identify that miR-122 can be a clinically practicable biomarker for cholestasis diagnosis with high sensitivity and specificity, further indicating that miR-122 might be a good and more reliable biomarker for cholestasis diagnosis.

The present study provides the first clinical evidence of circulating miR-122 as a biomarker of cholestasis-induced liver damage. However, research limitations such as small sample size do exist in our study. Therefore, additional investigations with larger cohorts of healthy people and patients are still needed to extensively evaluate the potential of miR-122 as a practical biomarker in comparison with other hepatic markers. On the other hand, although there is great interest in circulating miRNA as disease biomarkers, the characterization of many preanalytical and analytical parameters are still required in order to translate promising miRNAs into validated clinical tests. Unless these causes of imprecision are considered and mitigated, only miRNAs that are extremely up- or downregulated will be suitable as clinical biomarkers [29].

In summary, serum miR-122 levels were increased in cholestasis-induced liver injury in both BDL mouse models and patients with biliary calculi, perhaps through increased release of miR-122 from injured hepatocytes. Elevated liver-apecific miR-122 in serum may be a novel sensitive and specific biomarker for early detection of cholestatc liver injury in humans. Thus miR-122 might evolve as biomarkers in the diagnosis of biliary calculi.

## Supporting Information

Figure S1 **HE staining in sham-operated control mice and BDL mice.** The normal hepatic architecture was lost, extended necrotic areas were frequently observed and a marked ductal proliferation was present in BDL mice as compared with the control group (Sham). Inflammation, cholestasis and biliary epithelial cellular proliferation were present in the BDL mice.
(TIF)

Figure S2 **Bile-duct ligation led to elevated biliary epithelial cellular proliferation.** The brownish stained cells denote the positively stained ones. While CK-19 was found to be mainly expressed in ductal cells in the BDL mice liver (**B**), scarcely no CK-19 expression was observed in those of the sham-operated mice (**A**) (original magnification ×20).
TIF

Table S1 **Dynamic changes of serum ALT, AST, ALP, TBIL and DBIL concentrations during BDL.**
(DOC)

## References

[1] Eulalio A, Huntzinger E, Izaurralde E (2008) Getting to the root of miRNA-mediated gene silencing. Cell 132: 9–14. 18191211 10.1016/j.cell.2007.12.024

[2] Carthew RW, Sontheimer EJ (2009) Origins and mechanisms of miRNAs and siRNAs. Cell 136: 642–655. 19239886 10.1016/j.cell.2009.01.035 PMC2675692

[3] Williams AE (2008) Functional aspects of animal microRNAs. Cell Mol Life Sci 65: 545–562. 17965831 10.1007/s00018-007-7355-9 PMC11131689

[4] Mott JL, Kobayashi S, Bronk SF, Gores GJ (2007) Mir-29 regulates Mcl-1 protein expression and apoptosis. Oncogene 26: 6133–6140. 17404574 10.1038/sj.onc.1210436 PMC2432524

[5] Kanda T, Ishibashi O, Kawahigashi Y, Mishima T, Kosuge T, et al (2010) Identification of obstructive jaundice-related microRNAs in mouse liver. Hepatogastroenterology 57: 1013–1023. 21410023

[6] Roderburg C, Urban GW, Bettermann K, Vucur M, Zimmermann H, et al (2011) Micro-RNA profiling reveals a role for miR-29 in human and murine liver fibrosis. Hepatology 53: 209–218. 20890893 10.1002/hep.23922

[7] Huang J, Wang Y, Guo Y, Sun S (2010) Down-regulated microRNA-152 induces aberrant DNA methylation in hepatitis B virus-related hepatocellular carcinoma by targeting DNA methyltransferase 1. Hepatology 52: 60–70. 20578129 10.1002/hep.23660

[8] Huang Z, Huang D, Ni S, Peng Z, Sheng W, et al (2010) Plasma microRNAs are promising novel biomarkers for early detection of colorectal cancer. Int J Cancer 127: 118–126. 19876917 10.1002/ijc.25007

[9] Pineau P, Volinia S, McJunkin K, Marchio A, Battiston C, et al (2010) miR-221 overexpression contributes to liver tumorigenesis. Proc Natl Acad Sci U S A 107: 264–269. 20018759 10.1073/pnas.0907904107 PMC2806773

[10] Tsujiura M, Ichikawa D, Komatsu S, Shiozaki A, Takeshita H, et al (2010) Circulating microRNAs in plasma of patients with gastric cancers. Br J Cancer 102: 1174–1179. 20234369 10.1038/sj.bjc.6605608 PMC2853097

[11] Adachi T, Nakanishi M, Otsuka Y, Nishimura K, Hirokawa G, et al (2010) Plasma microRNA 499 as a biomarker of acute myocardial infarction. Clin Chem 56: 1183–1185. 20395621 10.1373/clinchem.2010.144121

[12] Wang GK, Zhu JQ, Zhang JT, Li Q, Li Y, et al (2010) Circulating microRNA: a novel potential biomarker for early diagnosis of acute myocardial infarction in humans. Eur Heart J 31: 659–666. 20159880 10.1093/eurheartj/ehq013

[13] Wang K, Zhang S, Marzolf B, Troisch P, Brightman A, et al (2009) Circulating microRNAs, potential biomarkers for drug-induced liver injury. PNAS 106: 4402–4407. 19246379 10.1073/pnas.0813371106 PMC2657429

[14] Zhang Y, Jia Y, Zheng R, Guo Y, Wang Y, et al (2010) Plasma microRNA-122 as a biomarker for viral-, alcohol-, and chemical-related hepatic diseases. Clin Chem 56: 1830–1838. 20930130 10.1373/clinchem.2010.147850

[15] Laterza OF, Lim L, Garrett-Engele PW, Vlasakova K, Muniappa N, et al (2009) Plasma microRNAs as sensitive and specific biomarkers of tissue injury. Clin Chem 55: 1977–1983. 19745058 10.1373/clinchem.2009.131797

[16] Chang J, Guo JT, Jiang D, Guo H, Taylor JM, et al (2008) Liver-specific microRNA miR-122 enhances the replication of hepatitis C virus in nonhepatic cells. J Virol 82: 8215–8223. 18550664 10.1128/JVI.02575-07 PMC2519557

[17] Esau C, Davis S, Murray SF, Yu XX, Pandey SK, et al (2006) miR-122 regulation of lipid metabolism revealed by in vivo antisense targeting. Cell Metab 3: 87–98. 16459310 10.1016/j.cmet.2006.01.005

[18] Jangra RK, Yi Mk, Lemon SM (2010) Regulation of Hepatitis C Virus Translation and Infectious Virus Production by the MicroRNA miR-122. J Virol 84: 6615–6625. 20427538 10.1128/JVI.00417-10 PMC2903297

[19] Sarasin-Filipowicz M, Krol J, Markiewicz I, Heim MH, Filipowicz W (2009) Decreased levels of microRNA miR-122 in individuals with hepatitis C responding poorly to interferon therapy. Nat Med 15: 31–33. 19122656 10.1038/nm.1902

[20] Bai SM, Nasser MW, Wang B, Hsu SH, Datta J, et al (2009) MicroRNA-122 inhibits tumorigenic properties of hepatocellular carcinoma cells and sensitizes these cells to sorafenib. J Biol Chem 284: 32015–32027. 19726678 10.1074/jbc.M109.016774 PMC2797273

[21] Lanford RE, Hildebrandt-Eriksen ES, Petri A, Persson R, Lindow M, et al (2010) Therapeutic silencing of microRNA-122 in primates with chronic hepatitis C virus infection. Science 327: 198–201. 19965718 10.1126/science.1178178 PMC3436126

[22] Jiang JX, Mikami K, Shah VH, Torok NJ (2008) Leptin induces phagocytosis of apoptotic bodies by hepatic stellate cells via a Rho guanosine triphos-phatase-dependent mechanism. Hepatology 48: 1497–1505. 18925608 10.1002/hep.22515 PMC2596754

[23] Lebovitz HE, Kreider M, Freed MI (2002) Evaluation of liver function in type 2 diabetic patients during clinical trials: evidence that rosiglitazone does not cause hepatic dysfunction. . Diabetes Care 25: 815–21. 11978674 10.2337/diacare.25.5.815

[24] Qin X, Li J, Cui Y, Liu Z, Zhao Z, et al (2012) Effect of folic acid intervention on ALT concentration in hypertensives without known hepatic disease: a randomized, double-blind, controlled trial. . Eur J Clin Nutr 66: 541–8. 22085872 10.1038/ejcn.2011.192

[25] Dufour DR, Lott JA, Nolte FS, Gretch DR, Koff RS, et al (2000) Diagnosis and monitoring of hepatic injury. II. Recommendations for use of laboratory tests in screening diagnosis and monitoring. . Clin Chem 46: 2050–68. 11106350 10.1093/clinchem/46.12.2050 PMC7110382

[26] Lagos-Quintana M, Rauhut R, Yalcin A, Meyer J, Lendeckel W, et al (2002) Identification of tissue-specific microRNAs from mouse. Curr Biol 12: 735–739. 12007417 10.1016/s0960-9822(02)00809-6

[27] Girard M, Jacquemin E, Munnich A, Lyonnet S, Henrion-Caude A (2008) miR-122, a paradigm for the role of microRNAs in the liver. J Hepatol 48: 648–656. 18291553 10.1016/j.jhep.2008.01.019

[28] Cheung O, Puri P, Eicken C, Contos MJ, Mirshahi F, et al (2008) Nonalcoholic steatohepatitis is associated with altered hepatic MicroRNA expression. Hepatology 48: 1810–1820. 19030170 10.1002/hep.22569 PMC2717729

[29] McDonald JS, Milosevic D, Reddi HV, Grebe SK, Algeciras-Schimnich A (2011) Analysis of circulating microRNA: preanalytical and analytical challenges. Clin Chem 57: 833–840. 21487102 10.1373/clinchem.2010.157198

