# Supplementary figures and images for: Circulating Liver-Specific miR-122 as a Novel Potential Biomarker for Diagnosis of Cholestatic Liver Injury
Source: PLoS One. 2013 Sep 27;8(9):e73133. doi: 10.1371/journal.pone.0073133 (PMC3785475; doi:10.1371/journal.pone.0073133)

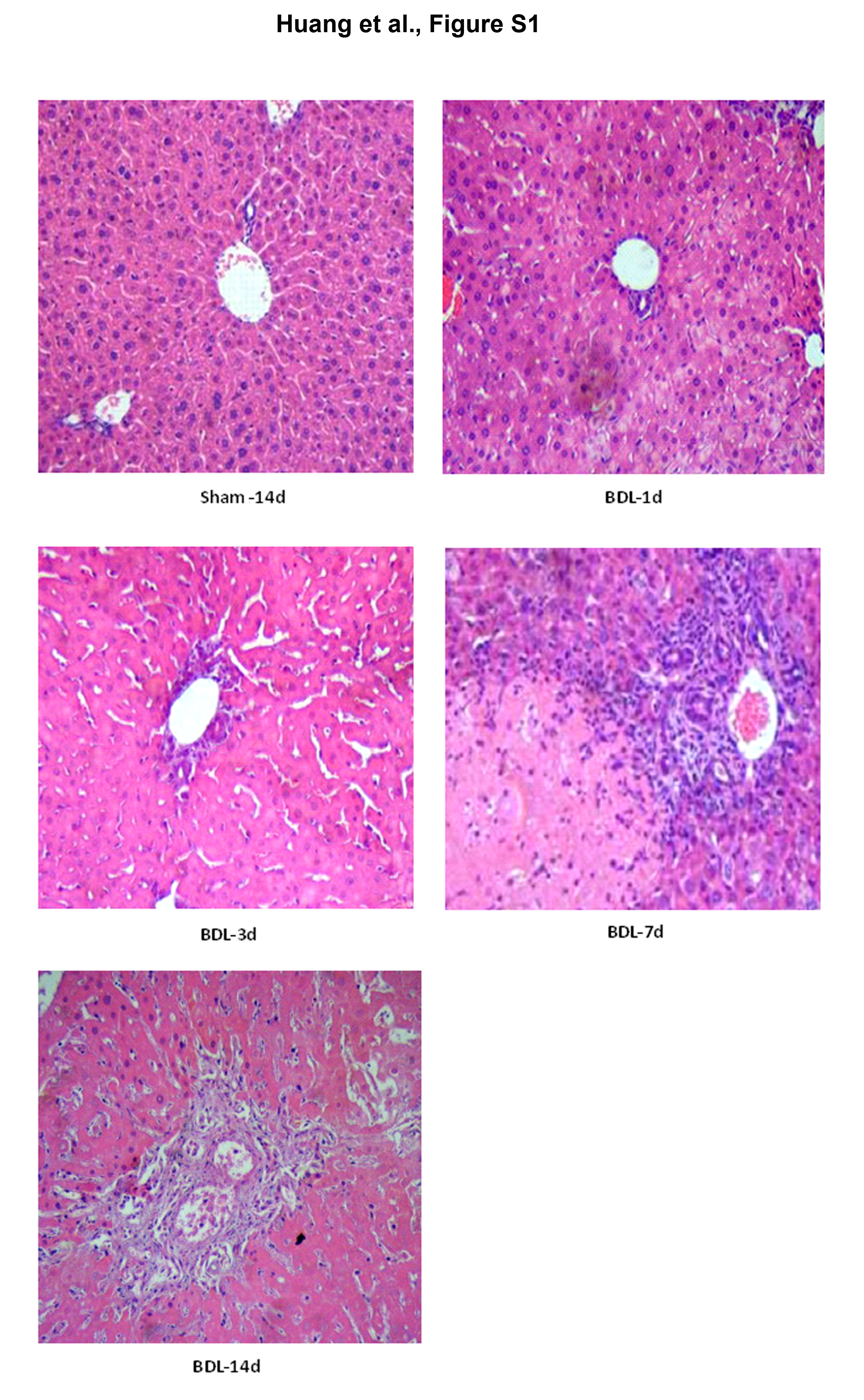

Supplement: Figure S1 — HE staining in sham-operated control mice and BDL mice. The normal hepatic architecture was lost, extended necrotic areas were frequently observed and a marked ductal proliferation was present in BDL mice as compared with the control group (Sham). Inflammation, cholestasis and biliary epithelial cellular proliferation were present in the BDL mice. (TIF) [file pone.0073133.s001.tif]

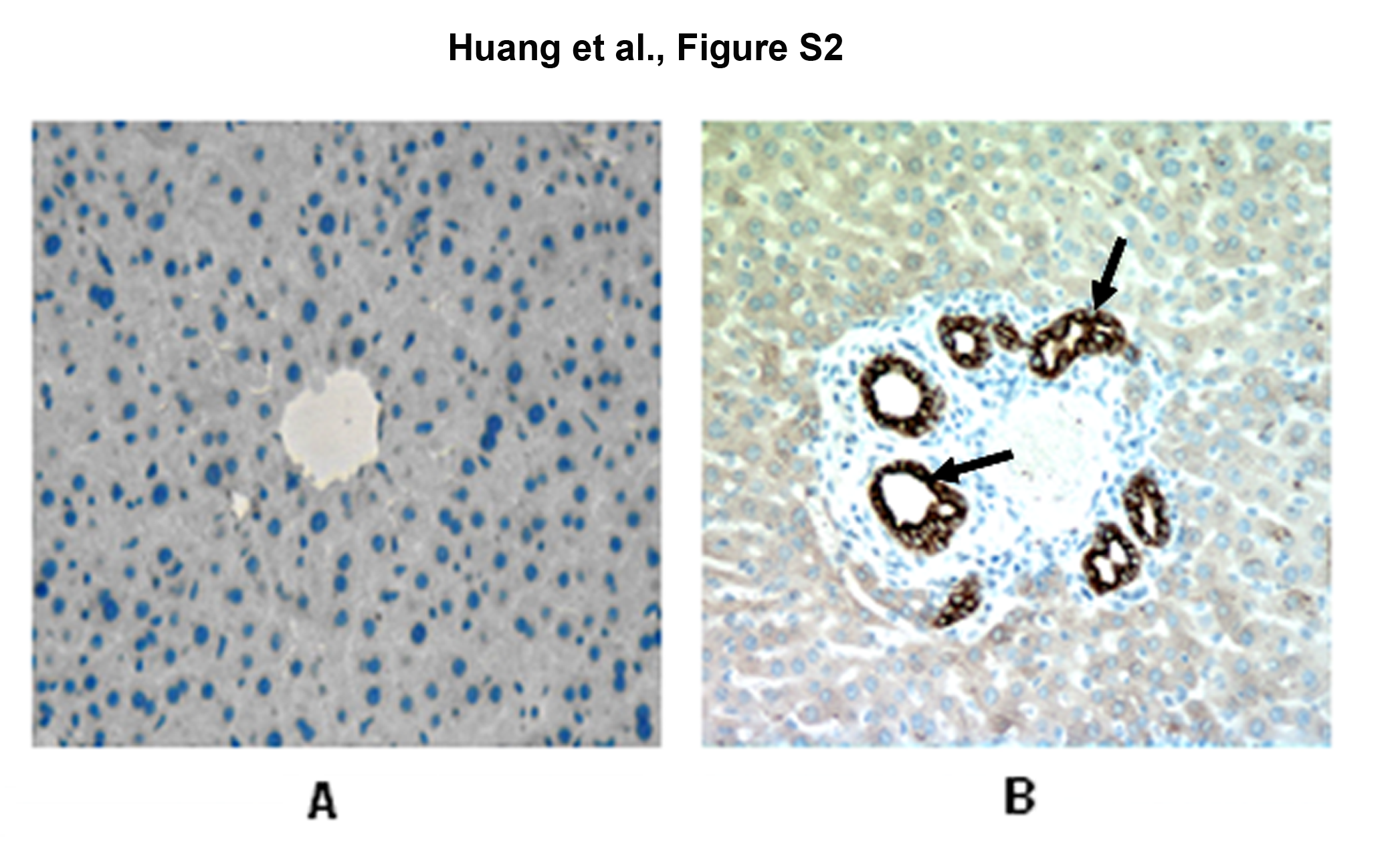

Supplement: Figure S2 — Bile-duct ligation led to elevated biliary epithelial cellular proliferation. The brownish stained cells denote the positively stained ones. While CK-19 was found to be mainly expressed in ductal cells in the BDL mice liver (B), scarcely no CK-19 expression was observed in those of the sham-operated mice (A) (original magnification ×20). TIF [file pone.0073133.s002.tif]
