# Supplementary material for: Circulating Liver-Specific miR-122 as a Novel Potential Biomarker for Diagnosis of Cholestatic Liver Injury
Source: PLoS One. 2013 Sep 27;8(9):e73133. doi: 10.1371/journal.pone.0073133 (PMC3785475; doi:10.1371/journal.pone.0073133)
Supplement: Table S1 — Dynamic changes of serum ALT, AST, ALP, TBIL and DBIL concentrations during BDL. (DOC) [file pone.0073133.s003.doc]

**Supplementary Tables**

**Table S**1 dynamic changes of serum ALT, AST, ALP, TBIL and DBIL concentrations during BDL

| Groups | ALT(U/L) | AST(U/L) | ALP(U/L) | TBIL  (μmol/L) | DBIL  (μmol/L) |
| --- | --- | --- | --- | --- | --- |
| Sham | 28.4±1.9 | 49.2±3.5 | 44.7±3.4 | 8.5±1.2 | 3.3±1.0 |
| BDL-1d | 352.2±29.1^*^ | 395.8±26.2^*^ | 402.6±28.4^*^ | 92.1±11.5^*^ | 74.8±9.3^*^ |
| BDL-3d | 865.0±92.2^*^ | 426.4±48.0^*^ | 455.3±35.2^*^ | 168.2±18.4^*^ | 135.7±12.5^*^ |
| BDL-7d | 395.8±50.0^*^ | 502.2±65.4^*^ | 596.7±52.1^*^ | 190.8±25.9^*^ | 157.6±17.2^*^ |
| BDL-14d | 656.4±83.2^*^ | 985.0±91.2^*^ | 1339±121.3^*^ | 320.6±29.1^*^ | 240.9±22.8^*^ |

*P＜0.05
